# Multilocus association mapping using generalized ridge logistic regression

**DOI:** 10.1186/1471-2105-12-384

**Published:** 2011-09-29

**Authors:** Zhe Liu, Yuanyuan Shen, Jurg Ott

**Affiliations:** 1 Department of Statistics, University of Chicago, 5734 S. University Avenue, Chicago, IL 60637, USA; 2 Department of Biostatistics, Harvard School of Public Health, 655 Huntington Avenue, Boston, MA 02115, USA; 3 Key Laboratory of Mental Health, Institute of Psychology, Chinese Academy of Sciences, 4A Datun Road, Beijing 100101, China

## Abstract

**Background:**

In genome-wide association studies, it is widely accepted that multilocus methods are more powerful than testing single-nucleotide polymorphisms (SNPs) one at a time. Among statistical approaches considering many predictors simultaneously, scan statistics are an effective tool for detecting susceptibility genomic regions and mapping disease genes. In this study, inspired by the idea of scan statistics, we propose a novel sliding window-based method for identifying a parsimonious subset of contiguous SNPs that best predict disease status.

**Results:**

Within each sliding window, we apply a forward model selection procedure using generalized ridge logistic regression for model fitness in each step. In power simulations, we compare the performance of our method with that of five other methods in current use. Averaging power over all the conditions considered, our method dominates the others. We also present two published datasets where our method is useful in causal SNP identification.

**Conclusions:**

Our method can automatically combine genetic information in local genomic regions and allow for linkage disequilibrium between SNPs. It can overcome some defects of the scan statistics approach and will be very promising in genome-wide case-control association studies.

## Background

In genome-wide association studies (GWAS), it is generally accepted that multilocus methods can obtain better power than single-locus approaches that test only one single-nucleotide polymorphism (SNP) at a time [1-3]. Among a large number of mathematical and statistical approaches considering many predictors simultaneously, scan statistics [4] serve as a useful multilocus analytical means for combining genetic information on multiple contiguous SNPs. In this method, the whole genome is scanned by a sliding window with estimated size, and the moving sum for each window is computed as the sum of suitable single-locus statistics. Then the scan statistic, defined as the largest moving sum, is calculated and its associated empirical *p*-value is evaluated by permutation tests.

Despite its remarkable advantages, the scan statistics method has two drawbacks that can restrict its practical use: (I) linkage disequilibrium (LD) within local genomic regions is not taken into account, which can result in an inflated type I error rate; (II) all contiguous SNPs within a genomic region (window) are selected simultaneously, which can bring excess noise and increase the false discovery rate (FDR).

During the last decade, various advances based on the framework of scan statistics have been developed. Zaykin *et al*. [5], Dudbridge *et al*. [6], and Yang *et al*. [7] proposed more effective and powerful test statistics within each sliding window and improved the sensitivity of scan statistics; Sun *et al*. [8] took into account the complex distribution of human genomic variation in the detection of causal chromosomal regions; Browning [9] and Li *et al*. [10] proposed a variable-sized sliding-window method based on Markov chain and regularized regression analysis. In their method, there is no need to specify a window size for haplotype tests, which makes it particularly useful in the investigation of association studies.

In this study, we propose a novel sliding window-based multilocus method for identifying a subset of susceptibility SNPs based on forward variable selection, using generalized ridge logistic regression (GRLR) for model fitness at each step. As a broader and generalized form of ridge logistic regression, GRLR can take advantage of prior information between any pair of SNPs and impose proper shrinkage penalty on each SNP within the genomic region of interest. Our method can automatically combine genetic information within local regions and select a subset of SNPs that best predict disease status, whose associated empirical significance level is assessed by permutation tests. We demonstrate by simulations and analysis of two published datasets that our method is highly informative and promising.

## Methods

### Generalized ridge logistic regression

Logistic regression (LR) is a common statistical method for looking into the dependency of a binomial response on a number of variables (predictors), whose general form is:

$$\log\left(\frac{p_{i}}{1-p_{i}}\right)=\beta_{0}+x_{i}^{T}\beta_{1},\;i=1,\;2,\ldots ,n,$$

where the *m*-dimensional vector *x*_*i *_represents the *i*th observation and *p*_*i *_is the probability of observing the *i*th outcome *y*_*i *_= 1. The regression coefficients parameter $\beta ={\left(\beta_{0},\beta_{1}^{T}\right)}^{T}$ can be estimated by maximizing the following log likelihood:

$$\begin{array}{c} L_{LR}\left(\beta \right)=\sum_{i=1}^{n}\left[y_{i}\text{log}(p_{i}\right)\\ +\left(\text{1}-y_{i}\right)\text{log}\left(\text{1}-p_{i}\right). \end{array}$$

Although logistic regression is very popular in case-control association analysis, it suffers from several shortcomings. If the number of SNPs in the regression model is larger than the number of observations, this method fails [1]. Moreover, with a large number of SNPs in the regression model, predictors can be highly correlated (high linkage disequilibrium), which can lead to further degradation of the model [11]. With the use of quadratic (*L*_2_) penalization, ridge logistic regression (RLR) [12] can overcome these disadvantages of logistic regression and increase the stability of model fitness. This method has recently been applied successfully in several biological investigations including accommodating linkage disequilibrium [13] and uncovering gene-gene interactions [11].

For the analysis discussed below, we consider generalized ridge logistic regression (GRLR), a broader and generalized form of ridge logistic regression, which amounts to maximizing the following penalized log likelihood:

$$\begin{array}{l} L_{GRLR}(\beta ;\lambda ,P)=\sum_{i=1}^{n}\left[y_{i}\log(p_{i}\right)\\ +(1-y_{i})\log(1-p_{i})]-\frac{1}{2}\lambda \beta_{1}{}^{T}P\beta_{1}, \end{array}$$

Where *p *is a nonnegative definite penalty matrix and *λ *is a positive scale constant, that is, a tuning parameter, which can be specified by cross-validation. The regression coefficients in the model are estimated using the *Newton*-*Raphson *iterative algorithm. The effective degrees of freedom and the variance of the coefficients can be approximated by estimators introduced in [14]. Then the Wald test can be applied to assign *p*-values to the regression coefficients.

Due to the feature of quadratic penalization that none of the coefficient estimators would be equal to zero in the shrinkage, GRLR cannot serve as an independent tool for model selection; however, the traditional forward selection procedure can be utilized, with the use of GRLR for model fitness in each round. In forward variable selection, we start with no predictor in the model and then add the one variable that leads to the best score. We continue adding variables one at a time until the score stops improving. In this study, we choose the AIC (Akaike Information Criterion) [15] as the scoring method in the variable selection procedure, which measures goodness of fit of a statistical model.

**Figure 1:**
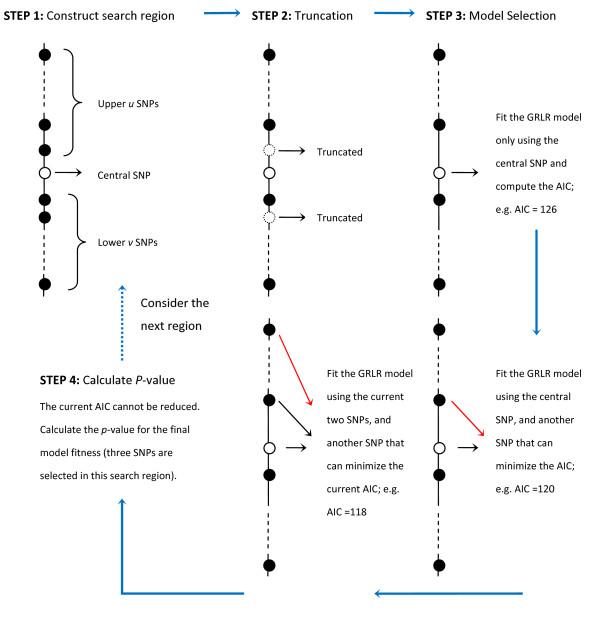
**Summary of steps in GRLR method**. Step 1: Construct search region; Step 2: SNP truncation; Step 3: Apply forward selection to the region, fitting the GRLR model in each iteration; Step 4: Calculate the *p*-value for final model and switch to next region.

As a proof of concept, we assume a sample of *M *individuals genotyped at *N *SNPs. For each individual, the three genotypes *AA, AB*, and *BB *on any given SNP are denoted by 0, 1, and 2, respectively, where the *B *allele is the minor allele, and an additive model is assumed. For the phenotype, the disease-affected status is denoted by 1, while the disease-unaffected status is denoted by 0. Let *x *be an *N *× *M *matrix containing the sample genotypes, where *N *is the number of SNPs and *M *is the number of individuals. Let *y *be an *M*-dimensional vector representing sample phenotypes. The procedure of our method is described below, and an intuitive flowchart is displayed in Figure 1.

Initially, we compute the *p*-values *P*_1_, *P*_2_, ..., *P*_*N *_for all the *N *SNPs, using logistic regression. For the *i*th SNP, where *i *= 1, 2, ..., *N*, we construct a search region with that SNP being in the center of the region. Let *u *and *v *denote the sizes (i.e. the numbers of SNPs) before and after this SNP, where *u *and *v *are always equal as long as the search region is entirely located in a chromosome. The constructed search region with the *i*th SNP as the central SNP is denoted by:

$$\begin{array}{l} G_{i}(u,v)=\{i-u,i-u+1,\\ \;\ldots ,i,\ldots ,i+v-1,i+v\}. \end{array}$$

To define the penalty matrix *p *in the GRLR model, we use the physical position information between pairs of SNPs in the search region, in order to impose different weights on different SNPs based on their distances to the center. The larger the distance between a SNP and the center, the more penalties would be placed on it. Other weighting functions for pairwise SNPs can also be implemented, such as the LD measurements *D *or *r*^2^. In this study, we only consider physical position information. Assume that the positions (base pairs) of SNPs in the region *G*_*i*_(*u*,*v*) are denoted by *W*_*i*_(*u, v*) = {*D*_*i-u*_, *D*_*i - u + 1*_, ..., *D*_*i*_, ..., *D*_*i + v + 1*_, *D*_*i + v*_}. Then the nonnegative definite penalty matrix *p *is defined by:

$$\begin{array}{l} P=\{diag[\sqrt{D_{i-u}-D_{i}},\sqrt{D_{i-u+1}-D_{i}},...,0,\\ \;\;\;...,\sqrt{D_{i+\nu -1}-D_{i}},\sqrt{D_{i+\nu }-D_{i}}]\}\\ \;\;\;/\sum_{j=i-u}^{i+\nu }\sqrt{D_{j}-D_{i}}. \end{array}$$

To avoid introducing excess noise, we do not use the information from all SNPs in each search region, because those with single-locus *p*-values too large (i.e. marginal effects too low) may contribute little to the power. To do this, we denote *t *as the *p*-value truncation threshold, and those SNPs with single-locus *p*-values larger than *t *will be excluded from the search region. In practical applications, the threshold *t *may be set somewhere from 0.05 to 0.10. After the truncation on *G*_*i*_(*u*,*v*), the adjusted search region is denoted by *T*_*i*_(*u*,*v*).

With the above configuration, we apply forward selection to each search region *Ti*(*u*,*v*), using GRLR for model fitting in each step. Denote *B*_*i*_(*u*,*v*) as the current best subset of SNPs for each step of the model selection procedure, which is empty at the beginning. We start by fitting the GRLR model using the central SNP (i.e. the *i*th SNP) as the only predictor, and calculating the corresponding AIC. Then we remove the central SNP from the search region, *T*_*i*_(*u*,*v*), and add it to *B*_*i*_(*u*,*v*). Next, the remaining SNPs in *T*_*i*_(*u*,*v*) are entered into the GRLR model one by one, along with the SNPs in *B*_*i*_(*u*,*v*) as predictors. We select the one SNP which can reduce the original AIC most. Then we add it to *B*_*i*_(*u*,*v*), remove it from *T*_*i*_(*u*,*v*), and update the current AIC. We repeat this procedure until none of the remaining SNPs in *T*_*i*_(*u*,*v*) can decrease the current AIC. Finally we investigate the last model and calculate the corresponding *p*-value $P_{\left\{E_{i}\right\}}$ for the model fitness, where the subset *E*_*i *_stands for the selected SNPs in this search region.

Considering all the search regions over the whole genome, our test statistic is defined as the minimum of all $P_{\left\{E_{i}\right\}}\text{s}$. To assign a global empirical *p*-value for the selected subset of SNPs, we use *L *permutations by randomly switching the case-control labels in the observed dataset. Considering the computational burden, we can construct search regions of interest using the top *S *SNPs whose *p*-values are smallest among all the *p*-values computed by the logistic regression. An alternative is to pre-define another truncation threshold and exclude the SNPs whose *p*-values exceed the threshold.

### Simulations

We apply our method to simulated data and compare its performance with that of five other methods in current use. For each replication, we generate a susceptibility genomic region with 21 SNPs (consisting of the central SNP, the upper *u *= 10 SNPs, and the lower *v *= 10 SNPs) under two scenarios. In Scenario (A), two SNPs within the region are disease-causing, while in Scenario (B), three SNPs are disease-causing. In both scenarios, one causal SNP is located at the center of genomic region while other causal SNPs are randomly located. We generate 100 independent individuals (50 cases and 50 controls), using the following logistic regression model:

$$\begin{array}{c} \log\left(\frac{p_{i}}{1-p_{i}}\right)=\beta_{0}+{\hat{\text{ a}}}_{1}x_{i1}+\\ \ldots +{\hat{\text{ a}}}_{k}x_{ik},\;i=1,\;2,\;\;\ldots ,\;n. \end{array}$$

In this model, the predictor *x*_*ij *_refers to the genotype of the *i*th individual at the *j*th causal SNP, where genotypes *AA, AB*, and *BB *are coded by 0, 1, and 2, respectively, assuming the *B *allele is the minor allele. In Scenario (A), *k *(the number of causal SNPs) equals 2, while in Scenario (B), *k *equals 3. The intercept *β*_o _is determined by requiring a disease prevalence of 0.05; other regression coefficients *β*_*j *_are set to 1.0.

Assume that each SNP within the region is derived from a multivariate normal distribution, and the variance-covariance matrix *Σ *is defined by: *Σ*_*pq *_= 1, when *p *= *q*; *Σ*_*pq *_= *r*, when |*p *- *q*| ≤ 5; *Σ*_*pq *_= 0, otherwise. The correlation coefficient *r *can be varied in simulations. For each SNP, we determine its genotype by its corresponding value generated from a multivariate normal distribution. If the value falls into the interval (-∞, qnorm((1 - *f*)^2^)), the genotype of this SNP is set to *AA*; if the value falls into the interval (qnorm(1 - *f *^2^), + ∞), the genotype of this SNP is set to *BB*; otherwise, the genotype of this SNP is set to *AB*. Here, *f *is the frequency of the *B *allele (i.e. minor allele frequency) and the function "qnorm" computes the quartile of standard normal distribution.

## Results

### Power calculations

We compare the performance of our method (GRLR) with that of logistic regression (LR), Fisher product method (FPM) [16], truncated product method (TPM) [5], lasso logistic regression (Lasso) [10,17,18] and elastic net (Enet) [19,20]. FPM uses a sliding window with fixed size to scan the genomic region, and for each window, it computes the sum of the logarithm of each *p*-value. Then the test statistic is defined as the minimum value over all windows. TPM is similar to FPM but it focuses on the *p*-values that are no more than a pre-fixed truncation threshold. In this simulation study, the truncation threshold for TPM method is set to 0.05 and the window size is set to 5 in both FPM and TPM methods. For the Lasso and Enet methods, all SNPs in the search region are entered into regression models and subsets of SNPs are selected. The tuning parameters in both methods are determined by cross-validation. For the GRLR method, the truncation threshold is also set to 0.05 and the tuning parameter *λ *is set to 1. We mimic the physical distances between different SNPs by constructing a simple mapping function that the position of the *b*^th ^SNP in the genomic region is set to *b*.

For each combination of these parameters, with minor allele frequencies *f *= 0.1, 0.3, or 0.5 and correlation coefficients *r *= 0.0, or 0.2, we carry out 500 replications and assess the power of various methods under both scenarios. To ensure that the type I error rate of each method is equal to 0.05, we utilize the following control procedure: for the FPM, TPM, and GRLR methods, 500 permutations are applied in each simulated replication. For the Lasso and Enet methods, since they often include extra terms and tend to have a high type I error rate [21,22], we apply 10 times ten-fold cross-validations for each generated replication. For each selected SNP in the observed data, its consistency is defined as the number of times that it occurs in 100 sub-datasets that contain 90 percent of observations. The consistency thresholds are determined under the null hypothesis to ensure the normal type I error rates.

For power considerations, the testing "success" can be defined by different strategies:

#### Scenario (A) (two causal SNPs)

• Strategy I: at least one of the two SNPs is detected

• Strategy II: both SNPs are detected

#### Scenario (B) (three causal SNPs)

• Strategy I: at least one of the three SNPs is detected

• Strategy II: at least two of the three SNPs are detected

• Strategy III: all three SNPs are detected

Tables 1 and 2 show the results of power simulations for the six methods under the conditions of Scenario (A) and Scenario (B), respectively. Considering all 30 conditions in the results, the averaged power for the six methods LR, FPM, TPM, Lasso, Enet, and GRLR, are equal to 0.373, 0.400, 0.394, 0.498, 0.539, and 0.558, respectively. Our GRLR method is the overall winner and Enet is second, being slightly less powerful than our method. Lasso performs worse than GRLR and Enet, but clearly outperforms the remaining three methods. FPM and TPM have quite similar power results, and LR ranks last. It is interesting to note that our GRLR method works very well under Strategies II and III, which indicates that our method is especially powerful in uncovering all causal SNPs within a given genomic region.

**Table 1:** Power calculation under Scenario (A)

*Strategy*	*Corr*	*MAF*	*Method*					
			
			*LR*	*FPM*	*TPM*	*Lasso*	*Enet*	*GRLR*
I	**0.0**	**0.10**	0.196	0.206	0.198	0.210	0.226	0.196
		**0.30**	0.638	0.620	0.602	0.688	0.670	0.650
		**0.50**	0.792	0.734	0.730	0.790	0.828	0.826
	**0.2**	**0.10**	0.184	0.216	0.194	0.194	0.204	0.206
		**0.30**	0.712	0.642	0.622	0.690	0.726	0.704
		**0.50**	0.860	0.794	0.788	0.902	0.888	0.870

II	**0.0**	**0.10**	0.008	0.078	0.070	0.032	0.052	0.124
		**0.30**	0.122	0.266	0.252	0.340	0.392	0.558
		**0.50**	0.354	0.304	0.306	0.526	0.624	0.768
	**0.2**	**0.10**	0.018	0.088	0.064	0.066	0.078	0.126
		**0.30**	0.202	0.296	0.300	0.364	0.458	0.608
		**0.50**	0.400	0.370	0.360	0.628	0.690	0.806

Results of power simulations for six methods under Scenario (A) (two causal SNPs). Two definitions of testing success, Strategy I (requiring that at least one of the two SNPs is significant) and Strategy II (requiring significance at both SNPs), are applied in comparisons; The number of replication runs is 500 for all the simulations. *Corr *= correlation coefficient; *MAF *= minor allele frequency.

**Table 2:** Power calculation under Scenario (B)

*Strategy*	*Corr*	*MAF*	*Method*					
			
			*LR*	*FPM*	*TPM*	*Lasso*	*Enet*	*GRLR*
I	**0.0**	**0.10**	0.302	0.346	0.320	0.330	0.388	0.310
		**0.30**	0.740	0.716	0.732	0.826	0.832	0.696
		**0.50**	0.906	0.888	0.900	0.932	0.940	0.872
	**0.2**	**0.10**	0.342	0.422	0.422	0.436	0.404	0.368
		**0.30**	0.814	0.846	0.832	0.838	0.854	0.784
		**0.50**	0.948	0.942	0.946	0.960	0.966	0.900

II	**0.0**	**0.10**	0.040	0.200	0.180	0.158	0.202	0.270
		**0.30**	0.280	0.478	0.490	0.630	0.704	0.680
		**0.50**	0.544	0.622	0.612	0.804	0.862	0.856
	**0.2**	**0.10**	0.058	0.256	0.264	0.210	0.238	0.314
		**0.30**	0.440	0.580	0.582	0.646	0.730	0.758
		**0.50**	0.700	0.672	0.678	0.858	0.914	0.890

III	**0.0**	**0.10**	0.002	0.022	0.016	0.044	0.066	0.100
		**0.30**	0.030	0.082	0.066	0.340	0.434	0.460
		**0.50**	0.140	0.084	0.076	0.558	0.640	0.656
	**0.2**	**0.10**	0.004	0.056	0.050	0.062	0.080	0.122
		**0.30**	0.124	0.088	0.090	0.324	0.422	0.490
		**0.50**	0.292	0.094	0.092	0.550	0.672	0.704

Results of power simulations for six methods under Scenario (B) (three causal SNPs). Three definitions of testing success, Strategy I (requiring that at least one of the three SNPs is significant), Strategy II (requiring that at least two of the three SNPs are significant), and Strategy III (requiring significance at all three SNPs), are applied in comparisons; The number of replication runs is 500 for all the simulations. *Corr *= correlation coefficient; *MAF *= minor allele frequency.

**Figure 2:**
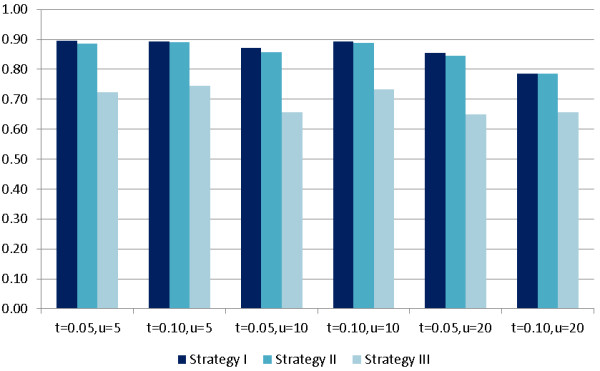
**Impact of different thresholds on power**. The effects of different truncation thresholds *t *= 0.05 or 0.10, and different sizes of regions 11, 21, or 41 (i.e. *u *= 5, 10, or 20) on power (y-axis). Only Scenario (B) (three causal SNPs) is considered, and the minor allele frequency is set to 0.50.

In our GRLR method, arbitrary choices of the truncation threshold and the size of the sliding window may become a potential problem. Thus we investigate the impact of different truncation thresholds *t *= 0.05 or 0.10, and different sizes of regions 11, 21, or 41 (i.e. *u *= 5, 10, or 20). We focus on Scenario (B) (three causal SNPs), and set the minor allele frequency to 0.5. Results are shown in Figure 2, where we can see that the power fluctuation caused by different choices of the two parameters is not large. It indicates that our method is not very sensitive to the choice of truncation threshold and the size of the sliding window.

We conducted additional simulations to test the performance of our method conditional on various choices of the tuning parameter *λ*. We consider Scenario (A) (two causal SNPs) and apply two definitions of testing success, Strategy I (requiring that at least one of the two SNPs is significant) and Strategy II (requiring significance at both SNPs). The correlation coefficient and the minor allele frequency are set to 0.0 and 0.30, respectively. The number of replication runs is 500 for all simulations. Results show that, when the tuning parameter *λ *equals 0.01, 0.1, 1.0, 10, and 100, the power of our proposed method under Strategy I equals 0.678, 0.650, 0.650, 0.640, and 0.672, respectively, while the power under Strategy II equals 0.600, 0.560, 0.558, 0.548, and 0.574, respectively. We can see that the power fluctuation caused by different values of *λ *is not large. It indicates that our method is not very sensitive to the selection of the tuning parameter, although it is unknown which selection can lead to the best result.

We further compared the performance of single-locus logistic regression and our proposed method by increasing the total number of SNPs in each simulated window to 200 and the number of causal SNPs to 10. For ease of calculation, only 100 permutations were applied in each of 200 replication runs. Simulation results show that, for logistic regression method, the proportion of times that it can successfully detect at least one causal SNP is 0.465, while the proportion of times that it can detect at least two causal SNPs is only 0.110; for our GRLR method, the proportion of times that it can successfully detect at least one causal SNP is 0.455, while the proportion of times that it can detect at least two casual SNPs is 0.445. These results indicate that our method is more powerful in discovering multiple disease variants.

### Analyzing published data

To further demonstrate our GRLR method and evaluate its practical applications in real dataset analysis, we apply it to two published genome-wide datasets: (1) a case-control dataset for heroin addiction [23] with approximately 10,000 SNPs and 200 individuals (100 cases and 100 controls), and (2) a case-control dataset for age-related macular degeneration (AMD) collected in Hong Kong [24,25] with approximately 100,000 SNPs and 223 individuals (96 cases and 127 controls).

For the heroin addiction data, we initially apply logistic regression on all the SNPs using the whole-genome data analysis toolset *PLINK *[26]. Table 3 shows the original *p*-values of the top 10 SNPs, as well as the adjusted *p*-values after applying the Bonferroni correction and 1000 permutations. From the result, none of the SNPs are statistically significant at threshold *α *= 0.05 after multiple test correction. To apply our GRLR method to the dataset, we construct genomic search regions based on the SNPs whose single-locus *p*-values are no larger than 0.05; the truncation threshold within each search region is also set to 0.05; the size on each side of the central SNP is set to 10; the tuning parameter *λ *in the GRLR model is set to 1.0. We use 1000 permutations to assign empirical *p*-values for the selected subset of SNPs. A summary of the analysis results is shown in Table 4, where the identified subset {rs1408830, rs965972} is statistically significant with a global empirical *p*-value of 0.027. It is interesting to note that using logistic regression or other single-locus methods, *p*-values for the two SNPs are 2.23 × 10^-04 ^and 5.06 × 10^-03^, neither of which is statistically significant at *α *= 0.05 after multiple testing corrections. In contrast, our method automatically combines the association information of the two SNPs and leads to a significant result.

**Table 3:** Results for heroin addiction data using logistic regression

*Rank*	*Chr*	*SNP rs#*	*Bp Position*	*Odds Ratio*	*Original P-value*	*Bonferroni Correction*	*Empirical P-value*
1	1	*rs*1408830	189929064	2.36	2.23E-04	1.0000	0.573
2	20	*rs*720010	7174248	2.04	4.20E-04	1.0000	0.841
3	13	*rs*950064	58410147	2.23	4.44E-04	1.0000	0.866
4	13	*rs*2016056	58410016	2.26	4.54E-04	1.0000	0.876
5	4	*rs*951299	99955054	0.48	6.68E-04	1.0000	0.955
6	11	*rs*1381784	42426136	2.33	8.68E-04	1.0000	0.984
7	5	*rs*2421057	158958294	2.19	8.70E-04	1.0000	0.984
8	17	*rs*1714984	12558426	2.26	9.37E-04	1.0000	0.986
9	9	*rs*3866796	15340199	0.50	9.48E-04	1.0000	0.986
10	4	*rs*1986513	126424833	0.17	1.34E-03	1.0000	0.998

Analysis results for the published dataset on heroin addiction using logistic regression. Odds ratios, original *p*-values, *p*-values after Bonferroni correction, and empirical *p*-values (derived from 1000 permutations) of the top 10 SNPs are listed.

**Table 4:** Results for both datasets using GRLR

*Dataset*	*Chr*	*Selected Subset of SNPs*	*Test Statistic*	*Empirical P-value*
heroin addiction	1	{rs1408830, rs965972}	1.26 × 10^-07^	0.027
AMD HK	10	{rs2736911, rs10490924, rs763720}	1.32 × 10^-11^	0.000

Analysis results for two published datasets using our GRLR method. Genomic search regions are constructed based on the SNPs whose single-locus *p*-values are no larger than a fixed threshold 0.05 for the heroin addiction data, and 0.01 for the AMD Hong Kong data; the truncation threshold within each search region is set to 0.05; the maximal length on each side of the central SNP is set to 10; the tuning parameter *λ *in GRLR model is set to 1.0. Empirical *p*-values are derived from 1000 permutations.

For the AMD Hong Kong data, after applying logistic regression, only the SNP rs10490924 is statistically significant, whose functional significance had been established experimentally [24]. Table 5 shows the original *p*-values of the top 10 SNPs, as well as adjusted *p*-values for multiple testing corrections. For our GRLR method, we construct search regions based on the SNPs whose single-locus *p*-values are no larger than 0.01. Other parameter configurations are the same as those applied in the first dataset. A summary of the results is shown in Table 4. Our method shows that the identified subset {rs2736911, rs10490924, rs763720} is statistically significant with an empirical *p*-value of 0 (evaluated by 1000 permutations). Based on this estimate, the 95% confidence interval for *p *extends from 0 through 0.003, which is considerably smaller than the published *p*-value of 0.027 (Table 4).

**Table 5:** Results for AMD Hong Kong data using logistic regression

*Rank*	*Chr*	*SNP rs#*	*Bp Position*	*Odds Ratio*	*Original P-value*	*Bonferroni Correction*	*Empirical P-value*
1	10	*rs*10490924	124204438	0.26	1.25E-09	0.0001	0.001
2	8	*rs*10504152	54292668	0.18	5.45E-06	0.4428	0.062
3	7	*rs*10499342	4340896	0.36	3.88E-05	1.0000	0.550
4	13	*rs*2011847	69496879	0.42	5.86E-05	1.0000	0.738
5	8	*rs*1377131	53838124	3.09	7.49E-05	1.0000	0.836
6	4	*rs*10520462	182400252	3.21	8.73E-05	1.0000	0.888
7	1	*rs*1564485	53333649	0.41	1.10E-04	1.0000	0.940
8	5	*rs*10521010	33931083	3.03	1.17E-04	1.0000	0.943
9	5	*rs*251610	65314237	0.45	1.55E-04	1.0000	0.980
10	20	*rs*1858597	95685	0.42	1.66E-04	1.0000	0.987

Analysis results for the published dataset on AMD Hong Kong using logistic regression. Odds ratios, original *p*-values, *p*-values after Bonferroni correction, and empirical *p*-values (derived from 1000 permutations) of the top 10 SNPs are listed.

## Discussion

Our method has the following advantages: (I) GRLR automatically combines association information of SNPs within each sliding window, and by truncating SNPs with low marginal effects and penalizing SNPs with a large distance from the center, our method can exclude excess noise and allow for linkage disequilibrium in local genomic regions; (II) we apply a forward model selection framework and fit the GRLR model at each step, since GRLR cannot serve as a means for variable selection. The results of power simulation and real datasets analysis indicate that this procedure works very well; (III) the global empirical *p*-value for the selected subset of SNPs is evaluated by permutation analysis, which properly handles the multiple testing problem and furnishes a valid type I error rate.

There are still some aspects in our GRLR method that can be improved in future work: (I) we do not consider the selection of the tuning parameter *λ *in this study. Cross-validation is an important tool for determining the tuning parameters in penalized regression approaches. It would be reasonable to determine *λ *by cross-validation, although it would introduce a higher computational burden; (II) the choices of window size and the truncation threshold in our method are arbitrary, which may reduce the stability of our method. Although simulation results show that the impact of these parameters on power is not large, a variable-size sliding-window procedure could be a better choice. Another way is to allow for the free variability of these parameters and then determine the best ones by permutations; (III) the Lasso and Enet methods can perform better than our proposed method in high-dimensional model selection, since they can deal with multiple variables simultaneously. In this case, the introduction of a truncation threshold is not quite necessary for the applications of these two methods. For power comparisons, however, we wanted to apply the same preselection procedure and the same truncation threshold to the Lasso and Enet methods. It is of our interest to investigate this further in future work; (IV) it is ideal to apply our region construction procedure to all SNPs. In this study, the search regions are constructed based on the SNPs whose marginal *p*-values do not exceed some pre-defined threshold; (V) it is of our interest to use the LD measurements or the combination of the LD and the physical distance information in constructing the weights for SNPs.

## Conclusions

In this study, we propose a new sliding window-based multilocus approach for detecting causal SNPs, which is based on forward model selection using generalized ridge logistic regression (GRLR) for model fitness at each step. Our method can overcome some defects of the scan statistics approach and provides a powerful procedure for identifying causal genomic region and mapping susceptibility genes in routine case-control association studies. In particular, because of its capability of automatically combining association information of multiple SNPs and its advantage on variable selection, our method can be a useful technique in the analyses of human complex diseases. Our software (available by request) is written in R [27], with the use of the *Design *Package [28] and *glmnet *Package [29].

## Competing interests

The authors declare that they have no competing interests.

## Authors' contributions

ZL and YS participated in the study design and the programming. JO contributed to the overall organization, reviewing and editing the manuscript. All authors read and approved the final manuscript.
